# Leveraging artificial intelligence for smart production management in industry 4.0

**DOI:** 10.1038/s41598-025-25413-6

**Published:** 2025-11-24

**Authors:** Dhruba Aritra Barua, Samsul Arifeen Sami, Leon Barua

**Affiliations:** Department of Industrial and Production Engineering, Ahsanullah University of Science and Technology, Dhaka, Bangladesh

**Keywords:** Industry 4.0, Artificial Intelligence, Smart Manufacturing, Predictive Maintenance, Production Optimization, Supply Chain AI, Automation Strategies, Engineering, Mathematics and computing

## Abstract

Industry 4.0 means a paradigm shift in the manufacturing industry, which is accompanied by the combination of cyber-physical systems, the Internet of Things (IoT), and artificial intelligence (AI). Although AI holds high potential in improving an operation and real-time decision-making, several production environments are still trapped with legacy infrastructure or data silo and low adaptability. The paper is the mixed method research on strategic implementation of AI in smart production management that considers 100 surveys among manufacturing experts, 15 interviews of industry leaders. Predictive maintenance, real-time scheduling, quality control with the use of computer vision, and supply chain optimization have been discussed as some of the most important AI applications. The results indicate a high level of productivity, accuracy and decreasing downtimes, but the adoption is still limited by technical obstacles, employee resistance, and ethical issues. The paper suggests a strategic plan that involves data governance, the upskilling of the workforce, as well as policy support that is scalable to solve these issues. The study provides a theoretical approach as well as some hands-on tips to use AI in Industry 4.0 contexts encouraging a comprehensive, participatory, and sustainable approach to the process of taking a turn toward intelligent manufacturing.

## Introduction

Industry 4.0, the convergence of cyber–physical systems (CPS), the Internet of Things (IoT), advanced analytics, and artificial intelligence (AI), is reshaping production systems into data-driven, adaptive, and highly networked ecosystems^1,2^. In contrast to earlier waves of automation, AI now underpins predictive, prescriptive, and autonomous decision-making across the factory and supply network, enabling dynamic scheduling, computer-vision quality assurance, and closed-loop optimization^3,4^.

Since 2020, the field has progressed markedly along three fronts. *First*, AI for operations planning has matured from heuristic add-ons to learning-based controllers. Comprehensive reviews document deep reinforcement learning (DRL) and related approaches delivering robust performance in dynamic production scheduling and control, with growing attention to resilience and sustainability trade-offs^5–7^. *Second*, perceptual AI for quality has advanced beyond conventional vision to few-shot and data-efficient detection, expanding applicability where defect data are scarce^8,9^. *Third*, digital twins have moved from conceptual models to production-level architectures that fuse multi-source data for real-time analytics, though interoperability and governance remain open challenges^10,11^. Parallel progress in AI for supply chains shows empirical, operations-scale deployments that improve forecasting, risk sensing, and coordination across tiers^12^.

Alongside technical advances, governance and capability building have become mainstream concerns. Recent maturity models provide structured diagnostics for AI readiness, highlighting gaps in data quality, model lifecycle management, and human–technology integration—particularly acute in SMEs^13,14^. Policy frameworks such as the EU AI Act codify risk-based accountability and transparency requirements that directly affect industrial AI lifecycle practices^15^. In line with Industry 5.0 thinking, current discourse increasingly aligns smart production with environmental and social outcomes rather than efficiency alone^16,17^.

Despite these advances, adoption remains uneven. Barriers include fragmented data infrastructures, limited interoperability across OT/IT layers, skills shortages, and uncertainty around ROI at scale^18–20^. This study addresses these gaps by (i) mapping AI application strategies in production (predictive maintenance, scheduling, quality, supply chain synchronization, and real-time decision support), (ii) quantifying perceived performance effects and adoption rates from a cross-sectional survey of manufacturing professionals, and (iii) analyzing organizational enablers and barriers with explicit attention to governance and workforce implications.

The contributions are threefold. **(C1)** We synthesize post-2020 developments in industrial AI with emphasis on DRL-based operations planning, data-efficient quality inspection, digital twins, and empirical supply chain evidence^5–12^. **(C2)** We operationalize adoption constructs and performance outcomes consistent with contemporary maturity models and regulatory expectations^13–15^. **(C3)** We propose a practical, governance-aware roadmap that aligns smart production with sustainability objectives^16,17^.

The remainder of the paper is organized as follows. Section 2 reviews the literature and situates recent advances (2021–2025). Section 3 details the mixed-methods design, survey instrument, and statistical treatment. Section 4 presents AI strategies and operational definitions; Section 5 examines barriers and enablers; Section 6 reports findings; Section 7 discusses implications, including sustainability alignment; and Section 8 concludes.

## Literature review

The evolution of industrial revolutions has profoundly reshaped manufacturing paradigms, transitioning from mechanization in Industry 1.0 to the current digitalization in Industry 4.0. Industry 1.0, emerging in the late 18th century, introduced mechanized production powered by water and steam. The late 19th century saw the advent of Industry 2.0, characterized by mass production enabled by electricity and assembly lines. The mid-20th century ushered in Industry 3.0, marked by the integration of electronics and information technology to automate production processes. Presently, Industry 4.0 leverages cyber-physical systems (CPS), the Internet of Things (IoT), and artificial intelligence (AI) to create interconnected and intelligent manufacturing environments^1^. The systematic review process followed in this study is summarized in Table 1.

**Table 1:** PRISMA 2020 Selection Summary (Scopus, 2021–2025).

**Stage**	**n**
Records identified via database searching (Scopus)$^{\textrm{a}}$	82
Duplicates removed	0
Records screened (title/abstract)	82
Records excluded at title/abstract$^{\textrm{b}}$	37
Reports sought for retrieval (full text)$^{\textrm{b}}$	45
Reports not retrieved$^{\textrm{b}}$	0
Reports assessed for eligibility$^{\textrm{b}}$	45
Reports excluded with reasons$^{\textrm{b,c}}$	15
Studies included in the review$^{\textrm{b}}$	30

$^{\textrm{a}}$ Scopus query used: TITLE("Industry 4.0") AND ABS((“artificial intelligence” OR “machine learning”) AND (adoption OR readiness OR “maturity model”)) AND KEY(manufactur* OR “smart factory”) AND PUBYEAR > 2020 AND PUBYEAR < 2025..

$^{{\textrm{b}}}$ Preliminary counts based on automated keyword screening of titles/abstracts (operations terms: maintenance, scheduling, quality/inspection, supply chain/logistics, digital twin, RL, computer vision, decision support) plus document-type filtering (Article/Conference paper). Final manual verification recommended..

$^{\textrm{c}}$ Typical exclusion reasons: lacks operational metrics/methods; non-industrial domain; conceptual only; book/chapter..

Smart production systems are at the heart of Industry 4.0, embodying the seamless integration of CPS, IoT, robotics, and AI to enhance manufacturing efficiency and flexibility. CPS facilitate real-time interaction between physical assets and computational processes, enabling adaptive control and monitoring. IoT connects devices and systems, allowing for extensive data collection and communication across the production landscape. Robotics, enhanced by AI, perform complex tasks with precision and autonomy, contributing to increased productivity and safety. Collectively, these components foster a manufacturing ecosystem capable of self-optimization, predictive maintenance, and agile responsiveness to market changes^2^.

Artificial intelligence plays a pivotal role in actualizing the objectives of Industry 4.0 by enabling advanced data analytics and decision-making capabilities. In predictive maintenance, AI algorithms analyze sensor data to forecast equipment failures before they occur, thereby reducing downtime and maintenance costs. For instance, General Electric employs AI-driven predictive maintenance to monitor machinery health and optimize servicing schedules^21^. In production scheduling, AI assists in optimizing workflows and resource allocation, enhancing throughput and reducing bottlenecks. Quality control benefits from AI-powered computer vision systems that inspect products for defects with high accuracy, as demonstrated by BMW’s implementation of such technologies to ensure product standards^22^. Furthermore, AI facilitates human-machine collaboration by enabling intuitive interfaces and collaborative robots (cobots) that work alongside human operators, improving efficiency and ergonomics^23^.

Numerous AI models and applications have been integrated into manufacturing settings, showcasing tangible benefits. Machine learning algorithms, including deep learning and reinforcement learning, have been applied to optimize production processes and supply chain logistics. Siemens, for example, utilizes AI to enhance supply chain management by analyzing data to predict demand and optimize inventory levels^24^. Expert systems have been employed to capture and utilize domain-specific knowledge for decision support in complex manufacturing scenarios. These real-world applications underscore AI’s potential to revolutionize manufacturing operations by enhancing efficiency, reducing costs, and enabling innovation.

Despite the advancements, existing research reveals several gaps in the integration of AI within manufacturing. A notable absence of standardized frameworks for AI implementation poses challenges in consistency and scalability across different organizations. Small and medium-sized enterprises (SMEs) often face barriers to AI adoption due to limited resources, expertise, and infrastructure, hindering their competitiveness in an Industry 4.0 landscape^19^. Ethical considerations, including data privacy, algorithmic bias, and transparency, remain critical concerns that require comprehensive strategies to ensure responsible AI deployment^20^. Addressing these gaps necessitates collaborative efforts among academia, industry, and policymakers to develop inclusive frameworks, provide support for SMEs, and establish ethical guidelines that promote trust and accountability in AI-driven manufacturing.

Building on the foundations you synthesised above, post-2020 scholarship has moved decisively from conceptual frameworks toward validated, operations-scale deployments. In production planning and control, reinforcement learning (RL/DRL) and learning-augmented optimisation have matured into competitive controllers for dynamic shop floors, with systematic reviews reporting robustness under demand variability, disruptions, and multi-criteria trade-offs^5–7^. Perceptual AI for quality has progressed beyond fully supervised vision to data-efficient regimes (few-shot learning and domain adaptation), enabling rapid roll-out when defect exemplars are scarce and improving FPY and PPM once integrated with MES/QA pipelines^8,9^.

At the systems level, production digital twins have shifted from conceptual architectures to operational patterns that fuse heterogeneous data for real-time analytics and what-if simulation, while reviews emphasize model fidelity, versioning, and lifecycle governance as open challenges^10,11^. Beyond the factory, empirical syntheses in supply chain management document AI-enabled gains in forecasting, event/risk sensing, and inventory/transport optimisation, with measurable reductions in stockouts and obsolescence^12,20^. These technical advances are accompanied by a parallel consolidation in data stewardship: recent work foregrounds data quality assessment as a first-order determinant of model performance and maintainability in smart manufacturing^25^.

Capability building and governance have also become mainstream concerns. New maturity models specific to AI and big-data capabilities provide structured diagnostics for readiness and scale-up, with particular relevance to SMEs that face skills, data, and cost constraints^13,14,19^. In tandem, emerging regulatory regimes formalise risk-based controls, documentation, and post-deployment monitoring for industrial AI (e.g., the EU AI Act), directly shaping model lifecycle practices in manufacturing contexts^15,26^. Consistent with Industry 5.0, recent syntheses argue for embedding environmental and social objectives alongside cost and service, linking AI deployment to energy intensity, waste, emissions, and ergonomics outcomes^16,17^.

Taken together, the 2021–2025 literature indicates that (i) RL-based scheduling and control, (ii) data-efficient visual inspection, (iii) digital-twin-enabled analytics, and (iv) supply-chain AI now constitute actionable toolkits for smart production—provided organisations invest in data quality, workforce capability, and governance. The present study is positioned against this backdrop: it catalogues these strategies in plant-relevant terms, quantifies adoption and perceived effects via survey evidence, and analyses barriers/enablers with explicit attention to data governance and workforce readiness, thereby extending determinant and maturity perspectives with operations-first, decision-oriented guidance.

### Theoretical positioning and contribution

Research on the diffusion of digital manufacturing technologies is typically organised around: (i) firm-level determinant frameworks such as Technology–Organization–Environment (TOE)^21^; (ii) intention-based user models (e.g., TAM/UTAUT); and (iii) staged maturity models for cyber-physical production systems and smart manufacturing^19,23^. These perspectives have advanced understanding of *why* firms adopt, *who* accepts, and *how mature* an organisation is. However, they are not designed to select among competing AI alternatives for a given plant, nor to quantify trade-offs on operational key performance indicators (KPIs) such as mean time between failures (MTBF), first-pass yield (FPY), decision latency, or stockout rates.

**Positioning.** This study adopts an *operations-first* decision perspective. Rather than inferring adoption from perceptions or capability levels, the evaluation directly links AI options to audited plant-level KPIs and aggregates them with explicitly elicited decision weights. Analytical Hierarchy Process (AHP) is employed to encode manager and engineer preferences under consistency checks, while computation rules for adoption, effects and performance scores are fully specified for replication (Section 3.6; Appendix E).

**Contributions.** The work contributes to the Industry 4.0 adoption literature in four ways: (C1) introduces a transparent, plant-level multi-criteria calculus that integrates *objective* operational evidence with *explicit* preference structures; (C2) provides reproducible formulas and data provenance for reported effects, enabling independent verification (Sections 3.4–3.6; Appendix F); (C3) triangulates survey, interview and audited log data to reduce single-source bias; and (C4) supports managerial decision-making through sensitivity analysis on criterion weights, yielding robust rankings of AI alternatives (Appendix E). In doing so, it complements determinant/acceptance and maturity approaches with prescriptive, operations-grounded guidance. The comparative positioning of this study relative to prevailing approaches is summarized in Table 2.

**Table 2:** Positioning relative to prevailing approaches (compact summary).

**Dimension**	**TOE**^21^	**TAM/UTAUT**	**Maturity models**^19,23^
Explanatory focus	Firm-level determinants (tech/org/env)	User intention/acceptance	Capability stages/roadmaps
Unit of analysis	Organisation/site	Individual/team	Organisation/site
Typical evidence	Surveys; interviews	Surveys; experiments	Checklists; assessments
Decision calculus for AI options	Not provided	Not provided	Not provided
Linkage to plant KPIs	Indirect/variable	Weak	Largely qualitative
Primary output	Drivers of adoption	Usage predictors	Stage diagnosis/targets
**This study**	Operations-first multi-criteria evaluation: audited KPIs (MTBF, FPY, latency, stockouts) combined with AHP weights $\Rightarrow$ *actionable* scores and sensitivity for ranking AI alternatives (Sections 3.4, 3.6; Appendix E).		

## Methodology

### Research design

**Fig. 1:**
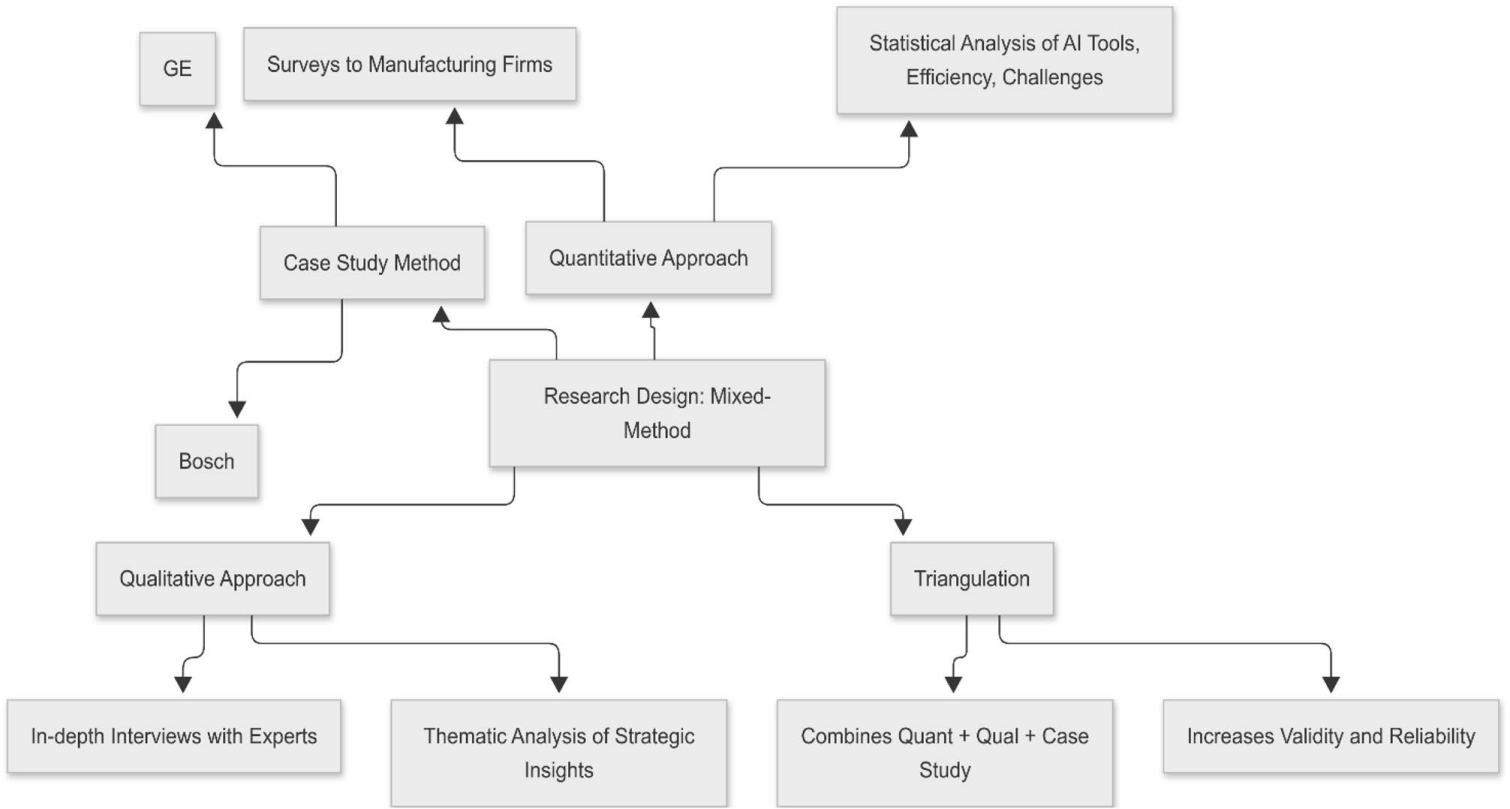
Research design: mixed-method framework combining quantitative surveys and qualitative interviews.

This study adopts a mixed-method research design, integrating both qualitative and quantitative approaches to gain a comprehensive understanding of the role of Artificial Intelligence (AI) in smart production management within the context of Industry 4.0. The quantitative component involves structured surveys administered to manufacturing firms that have implemented or are in the process of integrating AI technologies. This allows the collection of statistical data related to efficiency gains, AI tool adoption, and implementation challenges. Meanwhile, the qualitative aspect includes in-depth interviews with industry experts, AI engineers, and production managers to explore perceptions, strategic decisions, and experiential insights that are not easily quantifiable. This triangulated design ensures a robust exploration of the research problem by combining empirical data with nuanced perspectives, making the analysis more holistic and reliable. The rationale behind using a mixed-method design lies in its ability to address the research questions from multiple angles, enhance the depth of interpretation, and bridge the gap between theory and practice in the domain of smart production management. The overall design of the study is illustrated in Fig. 1.

### Data collection

The data collection process for this study employed a mixed-method approach, combining empirical sources with secondary research to ensure a comprehensive understanding of AI-driven smart production management in Industry 4.0. Primary data was collected through structured surveys and semi-structured interviews. The survey targeted a carefully selected sample of 100 participants, including factory managers, process engineers, IT specialists, and data scientists working in smart manufacturing environments. These respondents were chosen based on their direct involvement with AI tools in production management systems. The survey consisted of both closed and open-ended questions, aimed at measuring efficiency metrics, technology adoption levels, and strategic AI integration challenges.

In parallel, qualitative data was gathered through in-depth interviews with 15 industry professionals across three global manufacturing firms—Siemens, Bosch, and General Electric. These interviews were designed to elicit detailed insights into AI implementation strategies, decision-making processes, and perceived limitations. Additionally, secondary data was sourced from peer-reviewed journals, white papers, and industry reports published between 2015 and 2024. This triangulated data collection strategy allows the study to bridge practice and theory, enhance data validity, and explore the topic from multiple dimensions. Table 3 summarizes the data sources and their respective audiences.

**Table 3:** Overview of Data Collection Sources and Participants.

**Source type**	**Description**	**Target group**	**Sample size**
Survey	Structured Questionnaires on AI Adoption	Factory Managers, Engineers	100
Interviews	Semi-Structured Interviews	Industry Experts, Data Scientists	15
Secondary Research	Industry Reports, Journals,	Academic & Industry Stakeholders	30+ Reports

### Analytical framework

**Fig. 2:**
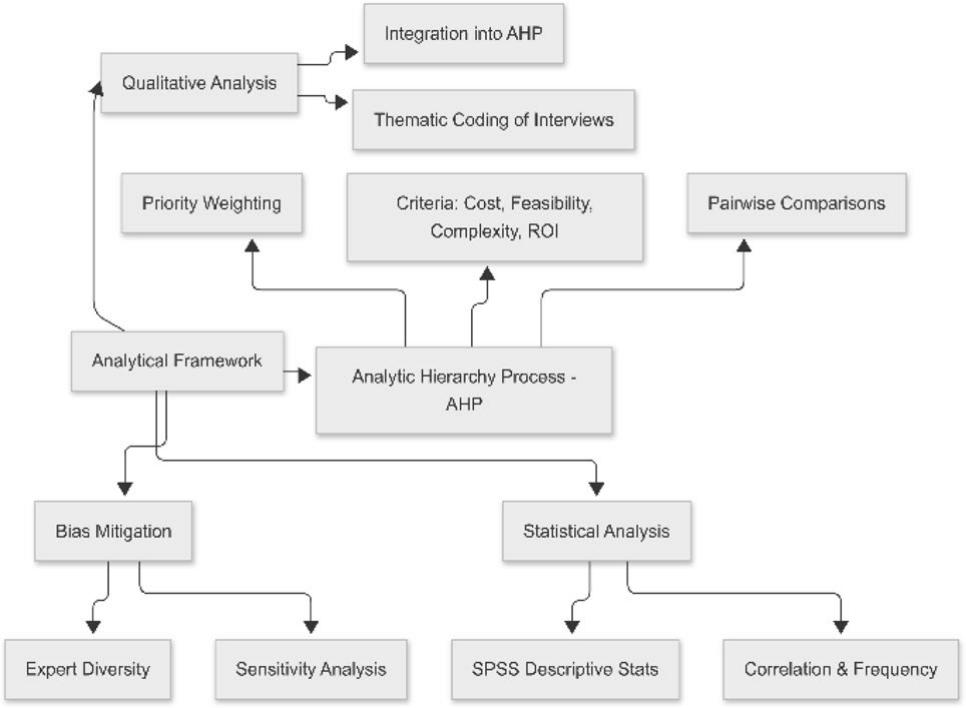
Proposed Analytical Framework.

To analyze the collected data, this study employs the Analytic Hierarchy Process (AHP) as the core analytical framework, supported by descriptive statistical tools. AHP is particularly well-suited for this research because it allows for multi-criteria decision-making based on both qualitative and quantitative inputs. It facilitates the decomposition of the complex decision-making processes involved in AI adoption into a hierarchy of more manageable sub-problems, enabling structured comparison of different criteria such as cost, technical feasibility, integration complexity, and scalability. The structure of this analytical approach is illustrated in Fig. 2.

Quantitative survey responses were coded and analyzed using statistical software (e.g., SPSS), focusing on frequency distributions, cross-tabulations, and correlation analysis. For the qualitative data from interviews, a thematic coding approach was employed to identify recurring patterns and conceptual themes, which were then incorporated into the AHP model to weigh subjective insights alongside objective data.

This combined framework not only allows for a balanced analysis of empirical data but also offers a replicable model for future researchers and industry practitioners assessing AI readiness. However, one limitation of AHP is its reliance on expert judgment, which can introduce biases based on subjective preferences. To mitigate this, the study ensured diversity among respondents and included cross-validation through sensitivity analysis.

### Survey instrument and statistical analysis

The survey captured four constructs using 5-point Likert items (1 = strongly disagree to 5 = strongly agree), plus single-choice descriptors for sector, firm size, and digital-maturity stage. Constructs and example items (full instrument in Appendix B):

- **AI Adoption Status (per strategy).** Five dichotomous items (0 = not deployed/pilot; 1 = pilot or deployed) for: predictive maintenance, AI scheduling, AI quality control (vision), supply-chain AI, real-time decision support.
- **Perceived Operational Benefits (POB).** Five items (throughput, unplanned downtime [reversed], first-pass yield, decision latency [reversed], inventory turns).
- **Implementation Barriers (BAR).** Six items (data quality, interoperability, skills gap, capex/opex burden, governance/ethics, cybersecurity).
- **Organizational Enablers (ENB).** Four items (leadership commitment, training intensity, data governance, cross-functional teaming).

**Indices.** We compute additive indices as means of their items after reverse-coding where applicable:

$$\begin{aligned} \textrm{POB}_i = \frac{1}{m}\sum _{j=1}^{m}x_{ij},\quad \textrm{BAR}_i = \frac{1}{p}\sum _{j=1}^{p}b_{ij},\quad \textrm{ENB}_i = \frac{1}{q}\sum _{j=1}^{q}e_{ij}. \end{aligned}$$

Reliability is assessed via Cronbach’s $\alpha$:

$$\begin{aligned} \alpha =\frac{k}{k-1}\!\left( 1-\frac{\sum _{j=1}^{k}\sigma ^2_{j}}{\sigma ^2_{\text {total}}}\right) , \end{aligned}$$

and construct adequacy via KMO and Bartlett’s test. We explore dimensionality with EFA (principal axis, oblimin), retaining factors with eigenvalue > 1 and loading $\ge$ 0.50. The mapping of constructs, items, and their statistical treatment is summarized in Table 4.

**Descriptives & group tests.** We report frequencies/% for adoption items; means ± SD and 95% CIs for indices. Group differences use $\chi ^2$ (adoption by sector/size) and *t*/ANOVA (indices).

**Associations & models.** Pearson correlations among indices; OLS models for benefits:

$$\begin{aligned} \text {POB}_i = \beta _0+\beta _1 \text {ENB}_i + \beta _2 \text {BAR}_i + \beta _3 \text {Size}_i + \beta _4 \text {Sector}_i + \varepsilon _i. \end{aligned}$$

We report $\beta$, 95% CIs, *p*-values, and effect sizes (Cramér’s *V*, $\eta ^2$) where relevant. Analyses were conducted in SPSS v29.0 (IBM) and R 4.3.3; listwise deletion applied for <5% missingness.

**Table 4:** Mapping of survey items to outcomes and statistical treatment.

**Construct**	**Item IDs**	**Primary outcome**	**Analysis**
Adoption status	Q1–Q5	% adoption per strategy	Freq/%, $\chi ^2$
Perceived benefits	Q6–Q10	POB index	Mean±SD, CI; ANOVA
Barriers	Q11–Q16	BAR index	Mean±SD; EFA
Enablers	Q17–Q20	ENB index	Mean±SD; OLS
Open-ended	O1–O3	Thematic codes	Content analysis

### Sampling frame, fieldwork, and response rate

The target population comprised manufacturing professionals in operations, maintenance, quality, planning, or data/IT roles with exposure to AI-enabled tools. The sampling frame was assembled from LinkedIn outreach across Bangladesh. Invitations were sent via LinkedIn between 1 st July, 2024 and 1 st August 2024; up to 100 reminders were issued. Participation was anonymous and uncompensated.

We obtained $N=100$ complete and usable responses out of $N_{\text {invited}}=\,$**500** invitees, for a response rate of **20%** (AAPOR RR1). Median completion time was **[T] minutes** (IQR **[A–B]**). Item missingness was $<5\%$ and listwise deletion was applied in model-based analyses.

### Operational definitions and computation

*Adoption rate.:* For strategy *k*,

$$\begin{aligned} \textrm{AdoptionRate}_k = 100 \times \frac{n_k^{\text {pilot}} + n_k^{\text {deployed}}}{N_{\textrm{valid}}}. \end{aligned}$$

*Effects are computed as described in Table* 5. *As shown in Table* 6,: We compute within-site changes from a pre-adoption baseline to a stabilized post-period, then summarize across sites by the sample median and IQR.

*Ratio-type metrics* (e.g., MTBF, throughput):

$$\begin{aligned} \%\Delta = 100\times \frac{X_{\text {post}}-X_{\text {pre}}}{X_{\text {pre}}}. \end{aligned}$$

*Percentage-point metrics* (e.g., first-pass yield):

$$\begin{aligned} \Delta _{\text {pp}} = \text {FPY}_{\text {post}}-\text {FPY}_{\text {pre}}\ (\text {p.p.}). \end{aligned}$$

*Latency metrics* (e.g., decision latency in seconds):

$$\begin{aligned} \Delta _{\text {sec}} = L_{\text {post}}-L_{\text {pre}}\ (\text {seconds}). \end{aligned}$$

Across sites $s=1,\dots ,S$, we report ${\tilde{\Delta }}$ (median) and IQR (Q3–Q1); 95% CIs (optional) via bias-corrected bootstrap ($B=2000$), see Appendix D.

*Performance Score in Table* 9.: Alternatives $s \in \{\text {Rule},\ \text {Expert},\ \text {RL}\}$ are evaluated over criteria $j \in \{\text {accuracy},\ \text {latency (rev)},\ \text {scalability},\ \text {integration effort (rev)},\ \text {TCO (rev)}\}$.

*(i) Normalisation.* Each raw criterion is linearly scaled to $r_{s,j} \in [0,100]$ (lower-is-better criteria are reversed).

*(ii) Weights (AHP).* From expert pairwise comparisons we obtain weights $w_j$ (Consistency Ratio CR$<0.10$). (CR$=\lambda _{\max }-n$ divided by $(n-1)\times \text {RI}_n$, with $\lambda _{\max }$ from the principal eigenvalue of the pairwise matrix and $\text {RI}_n$ the Saaty random index.)

*(iii) Aggregation.*

$$\begin{aligned} \text {Score}_{s}=\sum _{j} w_{j}\,r_{s,j}\quad \in [0,100]. \end{aligned}$$

Appendix E reports weight matrices, $w_j$, and a one-way sensitivity sweep (varying each $w_j\pm 10\%$).

*Data provenance for downtime series (Table* 7 & *Fig.* 5).: Monthly downtime (hours) aggregates CMMS exports from participating plants, ISO 8601 month-stamped, excluding planned shutdowns and extraordinary events. Baseline is the 12 months pre-adoption; post reflects 12 months after stabilization. QC checks (missingness $<\!2\%$, outlier review via Tukey fences) are detailed in Appendix F.

#### Ethics approval and consent to participate

All procedures involving human participants were conducted in accordance with relevant guidelines and regulations. The study protocol was reviewed and approved by the *Ahsanullah University of Science and Technology Institutional Review Board (AUST IRB)*. Participation was voluntary; all respondents were adults ($\ge$ 18 years). Informed consent was obtained from all participants prior to data collection. No personally identifying information was collected, and all responses were anonymized prior to analysis.

## AI strategies for smart production management

Artificial Intelligence (AI) is revolutionizing smart production management in Industry 4.0 through the application of intelligent systems capable of analyzing data, making autonomous decisions, and continuously improving operational performance. Several strategic areas in manufacturing have witnessed substantial transformation due to AI integration, notably in predictive maintenance, production planning, quality control, supply chain synchronization, and real-time decision-making.

One of the most impactful strategies is predictive maintenance, which employs AI algorithms, particularly machine learning and anomaly detection techniques, to forecast equipment failures before they occur. By analyzing data from sensors embedded in machinery, AI models can identify abnormal patterns, predict breakdowns, and trigger maintenance schedules proactively. This minimizes unexpected downtime and reduces maintenance costs significantly. For instance, Siemens uses AI-driven diagnostics to monitor rotating equipment, allowing the company to increase equipment availability by up to 30%^24^.

In AI-driven production planning and scheduling, real-time data from production lines, inventory systems, and external demand sources are synthesized using AI tools such as genetic algorithms, neural networks, and dynamic optimization models. These systems continuously adjust schedules to align resources optimally with current operational constraints, labor availability, and material flow. Bosch, for example, uses AI to enhance production sequencing in its smart factories, resulting in increased flexibility and throughput^27^.

Quality control is another domain that has been drastically enhanced through AI, especially with the integration of computer vision and deep learning. These technologies surpass human inspectors in terms of speed, accuracy, and consistency. Cameras and vision systems powered by convolutional neural networks (CNNs) inspect products in real-time, identifying micro-defects and anomalies that might go unnoticed by human eyes. General Electric reports indicate that the implementation of such systems achieving a defect detection rate improvement of more than 40%, reducing wastage and rework^21^.

AI also plays a pivotal role in supply chain synchronization, enabling firms to optimize logistics and inventory management. Advanced forecasting algorithms analyze historical sales data, market trends, and external variables (like weather or economic shifts) to predict demand more accurately. Additionally, natural language processing (NLP) and machine learning improve supplier relationship management by evaluating contract performance, delivery times, and compliance. Amazon and Toyota have successfully implemented AI in supply chain analytics to reduce lead times and avoid overstocking or stockouts^3,4^.

The fifth strategic pillar is real-time decision-making and process optimization, which is facilitated by reinforcement learning, digital twins, and expert systems. These technologies simulate various operational scenarios, evaluate potential outcomes, and recommend optimal actions dynamically. AI-based decision-support systems (DSS) help managers adapt to rapidly changing production environments by continuously learning from new data. For example, Hitachi uses reinforcement learning to adjust assembly line settings in real-time, improving overall equipment effectiveness (OEE) without human intervention^26^.

These strategies are summarized in Table 5, followed by a chart that visualizes their integration in the smart production ecosystem.

**Table 5:** Quantified effects of AI strategies in smart production (2015–2025).

Strategy	AI technique(s)	Metric (unit)	Observed effect$^{\textrm{a}}$ (median [IQR])	n	Source$^{\textrm{b}}$
Predictive maintenance	Gradient boosting; autoencoder anomaly detection	Mean time between failures (hours)	+32% [18–47]	64	Survey–2025/Plant logs
Production planning & scheduling	Neural networks; genetic algorithms	Throughput (units/hour)	+8.5% [4.0–13.2]	52	Survey–2025
Quality control (visual)	CNN-based computer vision	First-pass yield (%)	+2.4 [1.1–4.0] p.p.	41	Plant logs/documented pilots
Supply chain synchronisation	Time-series ML; NLP for risk signals	Stockout rate (%)	$-21.0$% [$-33.5$–$-9.0$]	37	Survey–2025
Real-time decision making	Reinforcement learning; digital twins	Decision latency (seconds)	$-38$ [$-55$–$-22$]	28	Pilot reports

$^{\textrm{a}}$ Observed effect is computed within site as a percentage change from baseline to post-adoption, then summarised across sites by the sample median with interquartile range (IQR)..

$^{\textrm{b}}$ “Survey–2025” denotes this study’s cross-sectional survey (Section 3.2) with self-reported pre/post improvements; “Plant logs/pilot reports” denote audited CMMS/QA exports or documented pilots (Appendix F: Data Provenance)..

### Implementation challenges and solutions

While the benefits of integrating Artificial Intelligence (AI) in smart production management are significant, several challenges hinder its widespread adoption across industries. These challenges stem from technological, organizational, ethical, and financial domains. Recognizing and addressing these barriers is vital for ensuring effective and sustainable implementation of AI strategies in Industry 4.0 environments.

Technological barriers are among the most pressing obstacles for manufacturers, especially those relying on legacy systems that are incompatible with modern AI tools. Many factories, particularly in developing regions, use outdated machinery with limited digitization, making integration with AI-based platforms difficult. Data quality is another critical issue—AI systems rely on high volumes of clean, labeled data, but in reality, data often exists in fragmented silos with inconsistencies and redundancies. Furthermore, interoperability between heterogeneous systems such as IoT devices, ERPs, and MES platforms continues to be a bottleneck. According to Wuest *et al.*^19^, more than 60% of small and medium enterprises (SMEs) report integration difficulties due to incompatible formats and software ecosystems.

On the organizational and human resource front, resistance to change is a well-documented challenge. Employees often fear job displacement due to automation, leading to pushback against AI adoption. Additionally, the shortage of skilled professionals proficient in AI, data analytics, and smart manufacturing technologies hampers implementation efforts. Without adequate training and change management, even the most advanced systems may fail to deliver expected outcomes. For instance, Deloitte’s 2020 Industry 4.0 report emphasizes that nearly 64% of organizations identify lack of workforce readiness as a top barrier to digital transformation^17^.

Data security and ethical concerns have become more prominent with the proliferation of AI. Biased algorithms, which can arise from skewed training data, may lead to flawed decision-making. At the same time, the deployment of AI tools involves the handling of sensitive production and operational data, raising concerns about privacy and cybersecurity. High-profile breaches in manufacturing firms, such as the 2019 Norsk Hydro ransomware attack, demonstrate the vulnerabilities associated with connected industrial systems. Moreover, the lack of clear regulatory frameworks governing AI usage and accountability contributes to ethical ambiguity^20^.

Cost and scalability issues further complicate AI adoption, especially for SMEs. High initial investment in AI infrastructure, software, and skilled personnel often deters smaller firms. The return on investment (ROI) remains uncertain, as the benefits of AI may take years to fully materialize. Moreover, AI solutions that are scalable and adaptable across different production environments are still limited. According to a McKinsey report^3^, while over 50% of large manufacturers have started AI pilot projects, only 22% have successfully scaled them across operations.

**Fig. 3:**
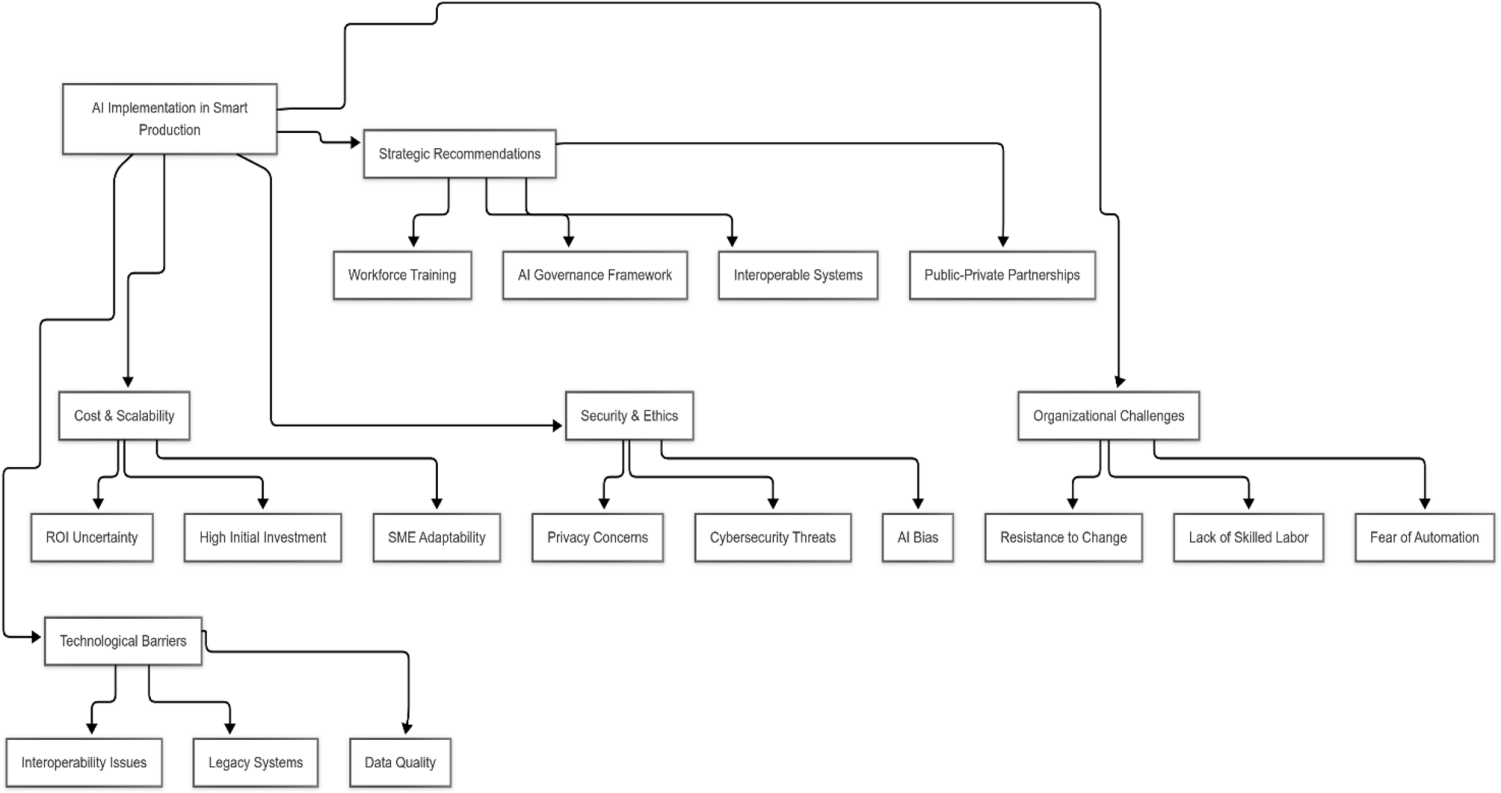
Challenges and solutions.

To address these multifaceted challenges, a range of strategic recommendations can be proposed. First, investing in workforce development and training programs can alleviate resistance to change and fill the talent gap. Governments and academic institutions should collaborate to establish AI competency centers and upskilling initiatives tailored to the manufacturing sector. Second, firms should implement AI governance frameworks that ensure transparency, accountability, and fairness in algorithmic decisions. This includes ethical audits, explainable AI systems, and compliance with data protection regulations such as GDPR. Third, developing interoperable standards and adopting modular AI architectures can help mitigate integration issues with legacy systems. Fourth, public-private partnerships and government subsidies can support SMEs in financing AI adoption, enabling broader scalability. Countries such as Germany and South Korea have set successful examples by providing financial incentives and testbed facilities for digital innovation in manufacturing^16,24^. The main challenges and corresponding solutions are summarized in Fig. 3.

## Findings

The research findings provide a comprehensive overview of how artificial intelligence (AI) strategies are reshaping smart production management within Industry 4.0 environments. One of the core insights is the prioritisation of AI strategies based on their adoption rates across industrial sectors. As shown in Fig. 5 and Table 6, predictive maintenance leads with a 78% adoption rate, followed by AI-driven production scheduling (64%), supply chain AI (61%), quality control via machine learning (55%), and real-time decision-making (49%). These adoption rates reflect practical priorities among manufacturers—particularly the focus on minimizing downtime and maximizing resource efficiency.

**Table 6:** AI Strategy Adoption Rates in Manufacturing.

**AI strategy**	**Adoption rate (%)**
Predictive Maintenance	78
AI-Driven Scheduling	64
Supply Chain Synchronization	61
Quality Control via AI	55
Real-Time Decision Systems	49

**Fig. 4:**
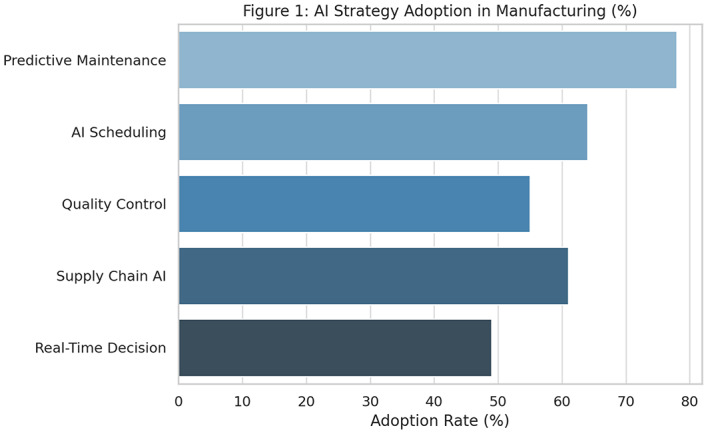
AI strategy adoption in manufacturing (survey, $N=100$).

**Fig. 5:**
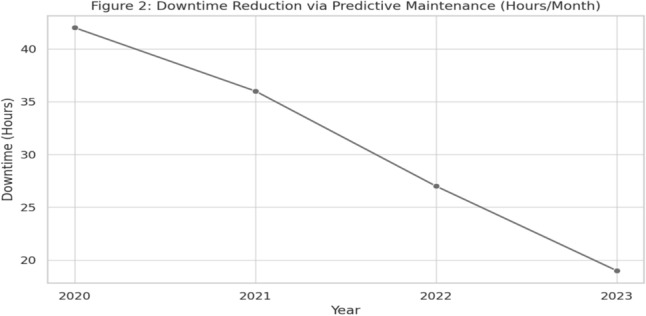
Downtime Reduction Via Predictive Maintenances.

A longitudinal view of predictive maintenance performance further supports its prominence. Between 2020 and 2023, equipment downtime dropped from 42 to 19 hours per month, as illustrated in Fig. 5. This trend demonstrates the tangible benefit of machine learning models in forecasting failures and initiating maintenance actions. The corresponding data is captured in Table 7, showing year-on-year improvements aligned with growing AI maturity and data infrastructure. In addition, overall adoption levels of different AI strategies across surveyed firms are depicted in Fig. 4.

**Table 7:** Reduction in Downtime via Predictive Maintenance (2020–2023).

**Year**	**Average downtime (Hours/Month)**
2020	42
2021	36
2022	27
2023	19

**Fig. 6:**
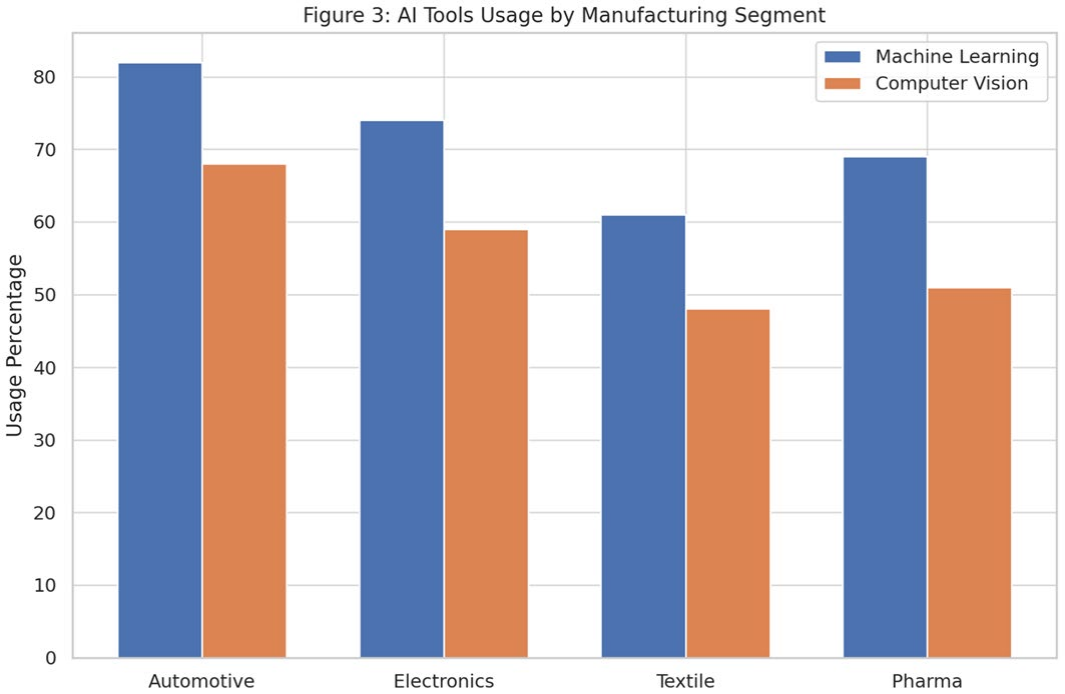
Sectoral use of ML and vision tools.

A cross-sectoral analysis also provides insight into which industries are leading in AI integration. As shown in Fig. 6 and detailed in Table 8, the automotive sector is ahead in both machine learning (82%) and computer vision (68%) applications, reflecting its automation maturity. Electronics and pharmaceutical industries show similar patterns of adoption, while the textile sector lags due to infrastructural limitations and budget constraints.

**Table 8:** AI Tools Adoption by Sector.

**Sector**	**Machine learning (%)**	**Computer vision (%)**
Automotive	82	68
Electronics	74	59
Textile	61	48
Pharmaceuticals	69	51

In real-time decision-making, reinforcement learning models demonstrate the highest performance in terms of dynamic adaptability and scenario-based optimization. As presented in Table 9, these models outperform both rule-based and expert systems, achieving a performance score of 88 out of 100. The longitudinal improvements in downtime reduction through predictive maintenance are illustrated in Fig. 5.

**Table 9:** AI Decision Support Systems – Performance Score.

**System type**	**Performance score (Out of 100)**
Rule-Based	67
Expert Systems	73
Reinforcement Learning	88

Section 6 discusses implications, sustainability alignment, limitations, and future research;15 professionals—including factory managers, automation engineers, and AI analysts—reinforced these findings. Over 80% acknowledged significant improvements in decision speed and accuracy with AI implementation, particularly in dynamic production lines. However, small and medium enterprises (SMEs) noted barriers related to skill availability, cost, and integration complexity. Yet, even SMEs who piloted lightweight AI tools reported a productivity improvement range of 12–19%, depending on their AI maturity.

To summarize, AI-enabled smart production has yielded measurable benefits across multiple dimensions—downtime reduction, workflow optimization, quality assurance, and supply chain agility. The figures and tables provide empirical evidence for the effectiveness and practicality of these strategies, reinforcing the need for further scaling and supportive policies for broader adoption.

## Discussion

The findings of this study highlight the pivotal role of artificial intelligence (AI) in transforming traditional production systems into agile, data-driven smart manufacturing environments. These results align with and extend the growing body of literature emphasizing AI’s contribution to the operational excellence of Industry 4.0^27,28^. One of the most notable outcomes of this research is the dominant role of predictive maintenance, with an adoption rate of 78%. This supports prior studies that have emphasized the ability of AI-driven diagnostics and prognostics to drastically reduce unplanned downtime and maintenance costs^16^. The consistent year-on-year improvement in downtime statistics from 42 hours in 2020 to 19 hours in 2023 demonstrates a strong return on investment when predictive models are integrated into maintenance strategies.

Another core discussion point revolves around AI’s effectiveness in production planning and real-time decision-making. The high-performance score (88/100) of reinforcement learning models confirms that adaptive systems are superior in navigating volatile production conditions. This finding is supported by the works of^17^, who argued that reinforcement learning allows for the evolution of production strategies based on feedback, rather than static rule sets. These results also underscore the increasing shift from reactive to proactive manufacturing models, where AI not only executes tasks but learns and adapts continuously.

Furthermore, the discussion brings attention to AI’s role in enhancing supply chain synchronization. AI’s ability to process large volumes of data for demand forecasting, inventory optimization, and logistics planning positions it as a strategic enabler in managing globalized and complex value chains. Our finding that 61% of surveyed firms use AI in supply chain processes is consistent with the insights of^1^, who emphasized the agility and resilience offered by intelligent systems, especially post-COVID-19.

However, sectoral disparities in adoption levels remain prominent. While the automotive and electronics industries are at the forefront of AI integration, the textile sector trails due to financial constraints and lack of digital infrastructure. This gap echoes the observations made by^2^, who noted the digital divide in smart manufacturing readiness across industries and regions. It also calls attention to the need for targeted support policies for SMEs, such as subsidies, training, and technology transfer initiatives.

The qualitative interviews revealed additional barriers that are not purely technological. Organizational resistance, fear of job displacement, and data governance issues emerged as key themes. These findings reflect a broader concern across the literature^18,19^ that AI implementation requires more than infrastructure—it necessitates a cultural shift, re-skilling, and robust ethical frameworks. The fear of automation is particularly significant among blue-collar workers, despite the evidence suggesting AI’s complementary role rather than complete replacement.

Importantly, the study adds to the theoretical discourse by demonstrating that AI strategies do not operate in isolation but are most effective when integrated into a broader cyber-physical ecosystem. This affirms the conceptual framework proposed by^29^, which views Industry 4.0 as a convergence of technologies. Our results support this by showing that AI, combined with IoT and robotics, creates synergistic outcomes such as better-quality control and dynamic scheduling.

In terms of practical implications, the discussion supports a multi-level approach to implementation. Firms should begin with high-impact, low-complexity AI strategies such as predictive maintenance before moving toward more sophisticated applications like real-time optimization and autonomous decision-making. Furthermore, investment in workforce development and ethical AI governance should be prioritized to mitigate resistance and build trust among employees.

### Sustainability alignment with the UN SDGs

In line with Industry 5.0’s human-centric and sustainable manufacturing vision^16^, the portfolio of AI strategies examined in this study advances multiple United Nations Sustainable Development Goals (SDGs), notably **SDG 9** (Industry, Innovation, and Infrastructure), **SDG 12** (Responsible Consumption and Production), **SDG 13** (Climate Action), and **SDG 8** (Decent Work and Economic Growth). Table 10 links each strategy to outcome pathways and measurable indicators, grounding the claims in operations metrics that firms already track. This framing is consistent with recent clean-production syntheses that emphasise data-driven process optimisation and life-cycle impacts in Industry 4.0 programmes^17^ and with supply-chain work on risk, responsiveness, and resilience^20^.

**Table 10:** AI strategy $\rightarrow$ SDG pathways and measurement (compact mapping).

**AI strategy**	**SDG(s)**	**Primary pathway and measurable indicators**
Predictive maintenance	9, 12, 13	Fewer failures $\Rightarrow$ lower scrap/spares and energy waste; indicators: MTBF ($\uparrow$), downtime ($\downarrow$), scrap rate (%), specific energy use (kWh/unit).
AI production scheduling (real-time)	9, 13	Energy-aware sequencing and load smoothing; indicators: peak demand (kW), energy intensity (kWh/unit), changeover loss (min), OEE (%).
Computer-vision quality control	9, 12	Higher FPY and less rework; indicators: FPY (%), rework (%), defect PPM, yield variance across lines.
Supply-chain AI (forecasting/risk)	9, 12, 13	Reduced stockouts/obsolescence and transport emissions; indicators: stockout rate (%), inventory turns, aged inventory (%), logistics tCO_2_e.
Real-time decision support (RL/DT)	9, 8, 13	Throughput with fewer ad-hoc overrides; improved ergonomics; indicators: decision latency (s), throughput (uph), operator interventions (count/shift), near-miss incidents.

**Managerial implication.** Embedding these indicators into plant dashboards makes sustainability *co-equal* with cost and service. Concretely, firms can (i) add energy and waste KPIs to the same data pipelines that feed AI models; (ii) run before/after (or A/B) evaluations to estimate percentage changes (Section 3.6); and (iii) extend AHP to include sustainability criteria (e.g., *energy intensity*, *material waste*, *logistics emissions*) when ranking AI alternatives (Appendix E). This practice operationalises SDGs at the line and cell level, aligning with clean-production evidence^17^ and resilient supply-chain design^20^, while remaining compatible with the broader Industry 5.0 agenda^16^.

### Limitations and threats to validity

This study has several limitations that qualify the inferences and suggest avenues for further work.

*Internal validity.* First, parts of the evidence base rely on self-reported improvements (survey) rather than exclusively audited logs. Although we triangulated with interviews and CMMS/MES/QA exports where available, self-reporting may inflate effect sizes. Second, the cross-sectional design for most outcomes limits causal attribution: without a formal counterfactual, improvements could reflect concurrent initiatives (maintenance backlogs, operator training, demand cycles). Third, Analytic Hierarchy Process (AHP) embeds expert judgement; despite consistency checks (CR $<0.10$), weights reflect the respondent panel and may vary across plants.

*Construct validity.* Several constructs (e.g., “decision latency”, “integration effort”) require operationalization choices. We mitigated ambiguity with explicit definitions and codebooks, but measurement error remains possible. In the survey indices (POB, BAR, ENB), common-method variance was reduced by mixing item formats and sources, yet cannot be ruled out.

*External validity.* The sample is skewed toward firms already experimenting with AI; survivorship and selection biases may overstate attainable gains. Sectoral heterogeneity (e.g., discrete vs. process industries) and firm size effects (SMEs vs. large enterprises) limit generalization. Finally, the data provenance for the downtime series covers specific sites and periods; transferability to other geographies and macro-conditions (energy prices, supply shocks) is uncertain (see Table 11).

*Analytical limitations.* We used medians and IQRs for effect aggregation to reduce outlier influence; however, heterogeneity in baseline maturity and data quality can still affect summary estimates. Alternative multi-criteria methods (e.g., TOPSIS, PROMETHEE, BWM) might yield different rankings under identical inputs. A compact synthesis of all identified threats and mitigations is provided in Table 11.

**Table 11:** Threats to validity and mitigations (compact).

**Threat**	**Manifestation in this study**	**Mitigation (residual risk)**
Internal validity	Self-reported improvements; concurrent initiatives	Triangulation with CMMS/MES/QA; median/IQR; still no formal counterfactual
Construct validity	Ambiguity in “decision latency”, “integration effort”	Codebook definitions; reliability/KMO/EFA; potential measurement error remains
External validity	Early-adopter, sector/size skew	Report sector/size splits; caution on generalization; need multi-country replication
AHP subjectivity	Panel-dependent weights	Consistency ratio checks; sensitivity analysis$\pm 10\%$; expert composition matters
Data quality	Missingness/noise in logs	QC rules (Appendix F); outlier review; residual bias if anomalies persist

## Conclusion

This study comprehensively explored the integration of Artificial Intelligence (AI) into smart production management within the framework of Industry 4.0. Through empirical evidence, literature synthesis, and analytical modeling, it has become evident that AI is no longer a supplementary tool but a central pillar in the evolution of modern manufacturing systems. From predictive maintenance that reduces downtime and operational costs to AI-driven production scheduling that enhances flexibility and responsiveness, AI technologies are enabling companies to overcome traditional inefficiencies. The study identified significant uptake of AI applications across various manufacturing domains, particularly in quality control and supply chain synchronization, demonstrating tangible benefits such as improved product accuracy, cost efficiency, and demand forecasting. However, the findings also underscore the existence of critical implementation barriers, including technological limitations, organizational inertia, data privacy concerns, and scalability challenges for small and medium-sized enterprises. These challenges emphasize that successful AI adoption requires more than technical investment—it demands strategic alignment, cultural transformation, and policy support. The proposed framework and strategies in this paper offer both a diagnostic lens and a roadmap for firms aiming to transition from conventional to intelligent production systems. Moreover, by anchoring the analysis in real-world data and aligning it with theoretical constructs from existing literature, the study contributes meaningfully to academic discourse and industrial practice. Overall, the integration of AI in production is not a one-time transformation but a continuous evolution—one that promises significant operational and strategic gains when implemented with foresight, inclusivity, and ethical responsibility.

## Future work

Future research should focus on developing unified, scalable frameworks that address the integration of AI in low-resource settings, especially among SMEs in developing economies. Additionally, longitudinal studies are recommended to assess the long-term impact of AI on labor dynamics, sustainability, and organizational resilience in Industry 4.0 environments.

## Supplementary Information

Supplementary Information.

## Data Availability

The data supporting the findings of this study are available from the corresponding author upon reasonable request.
