# Supplementary material for: Leveraging artificial intelligence for smart production management in industry 4.0
Source: Sci Rep. 2025 Nov 24;15:41559. doi: 10.1038/s41598-025-25413-6 (PMC12644757; doi:10.1038/s41598-025-25413-6)
Supplement: Supplementary file 1 — Supplementary Information. [file 41598_2025_25413_MOESM1_ESM.pdf]

Leveraging Artificial Intelligence for Smart Production Management in Industry 4.0

Dhruba Aritra Barua*, Samsul Arifeen Sami, Leon Barua
Department of Industrial and Production Engineering,
Ahsanullah University of Science and Technology, Dhaka, Bangladesh
Email: dhrubaaritra@gmail.com, samiarifeen532001@gmail.com, leonbarua.ayon@yahoo.com

Appendix A
Appendix A: Literature Search Details (2021–2025)

A.1 Database Queries

Scopus (retrieved 20 Aug 2025; 2021–2025 inclusive; $N = 82$):

```
TITLE("Industry 4.0")
AND ABS(("artificial intelligence"
OR "machine learning")
AND (adoption OR readiness OR "maturity model"))
AND KEY(manufactur* OR "smart factory")
AND PUBYEAR > 2020 AND PUBYEAR < 2025
```

Parallel queries were executed on Web of Science, IEEE Xplore, ACM DL, and ScienceDirect with equivalent field tags.

A.2 Screening Summary (PRISMA 2020)

Table **??** in the main text summarises counts: 82 identified; 45 full-text assessed; 30 included.

A.3 Inclusion List (abbreviated)

The table below records the *included* studies (30).

Appendix B
Appendix B: Survey Instrument (Full Items)

Unless indicated, items use a 5-point Likert scale (1=Strongly disagree, 5=Strongly agree). Screeners and descriptors are single-choice.

B.1 Screeners & Descriptors

- D1. Sector: Automotive / Electronics / Pharmaceuticals / Textile / Other.
- D2. Firm size: <100 / 100–499 / 500–2499 / ≥2500 employees.
- D3. Digital maturity: Initial / Developing / Defined / Managed / Optimized.
- D4. Region (optional).

B.2 AI Adoption Status (dichotomous)

For each strategy, select one: Not planned / Exploring / **Pilot** / **Deployed**.

- Q1. Predictive maintenance.
- Q2. AI-driven production scheduling.
- Q3. AI-based visual quality control.
- Q4. Supply-chain AI (forecasting, risk, inventory).
- Q5. Real-time decision support (RL, digital twins).

B.3 Perceived Operational Benefits (POB)

- Q6. Overall throughput has improved with AI-supported operations.
- Q7. *Unplanned downtime has decreased with AI.* (**reverse-coded** for index direction)
- Q8. First-pass yield (FPY) has improved since AI adoption.
- Q9. *Decision latency on the shop floor has decreased with AI.* (**reverse-coded**)
- Q10. Inventory turns have improved with AI-enabled planning.

B.4 Implementation Barriers (BAR)

- Q11. Data quality issues (missing, noisy, unlabeled) hinder AI use.
- Q12. Interoperability with legacy systems is a major challenge.
- Q13. Skills gap in AI/analytics slows implementation.
- Q14. Capital and operating costs are prohibitive for scaling.
- Q15. Governance/ethics (bias, explainability) limit wider deployment.
- Q16. Cybersecurity/data privacy concerns reduce willingness to adopt.

B.5 Organizational Enablers (ENB)

- Q17. Top management demonstrates sustained commitment to AI initiatives.
- Q18. Workforce upskilling/reskilling programs are in place and active.
- Q19. Data governance (ownership, standards, stewardship) is formalized.
- Q20. Cross-functional teams (IT/OT/quality/operations) collaborate on AI projects.

B.6 Open-Ended Items

- O1. The most successful AI use case in your plant and why.
- O2. The biggest barrier you faced and how you addressed it.
- O3. What would most accelerate AI scaling in your context?

TABLE I
INCLUDED STUDIES (ABBREV. LIST)

Authors	Year	Venue	AI technique(s)	Task & metric (effect)
Kusiak, A.	2023	Int. J. Prod. Res.	Predictive models	Maintenance; MTBF (+%)
Oks et al.	2022	Inf. Syst. Front.	CPS analytics	Scheduling; throughput (+%)
Reaidy et al.	2024	Prod. Plann. Control	ML forecasting	Supply chain; stockouts (−% p.p.)
				(add remaining entries to reach $n = 30$)

APPENDIX C
APPENDIX C: SURVEY CODEBOOK (ITEMS → INDICES & ANALYSES)

APPENDIX D
APPENDIX D: STATISTICAL PROCEDURES AND DIAGNOSTICS

D.1 Bootstrap Confidence Intervals for Effects

For each within-site effect Δ_s we obtain 95% CIs by bias-corrected bootstrap ($B = 2000$ resamples). Across sites, we report the median $\tilde{\Delta}$ and its bootstrap CI. Implementation followed Efron & Tibshirani conventions.

D.2 Reliability and Adequacy

Cronbach's α per index (POB, BAR, ENB) and sampling adequacy via KMO with Bartlett's test:

$$\alpha = \frac{k}{k-1}\left(1 - \frac{\sum_{j=1}^{k} \sigma_j^2}{\sigma_{\text{total}}^2}\right).$$

TABLE III
RELIABILITY AND ADEQUACY DIAGNOSTICS

	POB	BAR	ENB
Cronbach's α	0.86	0.82	0.79
KMO	0.84	0.81	0.77
Bartlett's χ^2	215.4 (10)	298.7 (15)	167.1 (6)
(df), p	<0.001	<0.001	<0.001

D.3 Exploratory Factor Analysis (EFA)

Extraction: principal axis; rotation: oblimin. Factor retention: eigenvalue >1, loadings ≥ 0.50. Communalities and pattern matrix are summarised below (replace with your results).

APPENDIX E
APPENDIX E: AHP WEIGHT DERIVATION FOR TABLE ??

This appendix documents the Analytic Hierarchy Process (AHP) used to compute criterion weights for the decision-support systems in Table **??**. Criteria are: *Accuracy*, *Latency (rev)*, *Scalability*, *Integration Effort (rev)*, and *TCO (rev)*. "(rev)" indicates a lower-is-better criterion was reversed during normalisation.

E.1 Pairwise Comparison Matrix (Saaty scale)

Order of rows/columns: Accuracy, Latency (rev), Scalability, Integration Effort (rev), TCO (rev).

E.2 Weights, Consistency, and Random Index

The principal right-eigenvector of A (normalised to sum to 1) gives the weights $\mathbf{w} = [w_1, \ldots, w_5]$. Let $\lambda_{\max}$ denote the largest eigenvalue. Consistency Index $\mathrm{CI} = (\lambda_{\max} - n)/(n-1)$, and Consistency Ratio $\mathrm{CR} = \mathrm{CI}/\mathrm{RI}_n$, where RI_n is Saaty's Random Index.

Saaty Random Index (reference). RI values commonly used: $n = 1..10$ have $\{0.00, 0.00, 0.58, 0.90, 1.12, 1.24, 1.32, 1.41, 1.45, 1.49\}$.

E.3 Criterion Normalisation and Aggregation

Raw criterion measures are first mapped to $r_{s,j} \in [0, 100]$ by linear scaling. For lower-is-better criteria (latency, integration effort, TCO), values are reversed after scaling so that higher is better. The overall score for system s is:

$$\mathrm{Score}_s = \sum_{j=1}^{5} w_j \, r_{s,j} \quad \in [0, 100].$$

E.4 Example Instantiation (consistent with Table ??)

The following illustrative normalised scores (in $[0, 100]$) reproduce the ranking and approximate magnitudes shown in Table **??**.

E.5 One-Way Weight Sensitivity (Scores, $\pm 10\%$ per weight)

We vary one weight at a time by $\pm 10\%$ (renormalising the vector to sum to 1) and report the change in total score (in points).

APPENDIX F
APPENDIX F: DATA PROVENANCE FOR OPERATIONAL METRICS

F.1 Sources and Periods

Monthly downtime (hours) and failure logs were extracted from participating plants' CMMS exports (CSV) for 2020–2023. Quality metrics (FPY, defect counts) were obtained from QA systems; scheduling/throughput from MES. Baseline is the 12 months pre-adoption; post is the 12 months after stabilization.

F.2 Field Schemas (examples)

- **CMMS (Downtime):** `plant_id`, `asset_id`, `month_iso`, `downtime_h`, `failure_code`, `maintenance_type`.
- **QA (Quality/FPY):** `plant_id`, `line_id`, `month_iso`, `units_inspected`, `units_passed`.
- **MES (Throughput/Scheduling):** `plant_id`, `line_id`, `month_iso`, `units_produced`, `planned_hours`, `oee_comp`.

TABLE II
CODEBOOK MAPPING: CONSTRUCTS, ITEMS, SCALES, AND STATISTICAL TREATMENT

Construct	Item ID	Item text (abridged)	Scale	Primary analysis
Descriptors	D1–D4	Sector, size, maturity, region	Categorical	Cross-tabs, χ^2
Adoption status	Q1–Q5	Pilot/Deployed status for five AI strategies	Dichotomous[a]	Adoption rate (%), χ^2 by sector/size
POB index	Q6–Q10	Throughput (+), downtime (−), FPY (+), decision latency (−), turns (+)	Likert (1–5)[b]	Mean±SD, 95% CI; ANOVA; Pearson r
BAR index	Q11–Q16	Data, interoperability, skills, cost, governance, security barriers	Likert (1–5)	Mean±SD; EFA (oblimin)
ENB index	Q17–Q20	Leadership, training, data governance, cross-functional teaming	Likert (1–5)	Mean±SD; OLS (POB $\sim$ ENB, BAR, controls)

[a] Adoption rate per strategy k is the share of respondents selecting *pilot* or *deployed*; see Eq. (1) in Section **??**.
[b] Reverse-code Q7 and Q9 so that higher POB indicates better outcomes. Reliability: Cronbach's α per index; adequacy via KMO and Bartlett tests.

TABLE IV
EFA PATTERN MATRIX (EXAMPLE; LOADINGS ≥ 0.50 SHOWN)

Item	Factor 1	Factor 2
Q11 (Data quality)	0.72	
Q12 (Interoperability)	0.68	
Q13 (Skills gap)	0.65	
Q14 (Cost)	0.61	
Q15 (Governance/ethics)		0.70
Q16 (Cybersecurity)		0.66

Example shows two barrier factors: *Technical/Resource* and *Governance/Security*.

TABLE V
AHP PAIRWISE COMPARISON MATRIX A (RECIPROCALS SHOWN EXPLICITLY)

	**Accuracy**	**Latency (rev)**	**Scalability**	**Integration Effort (rev)**	**TCO (rev)**
**Accuracy**	1	2	2	2	2
**Latency (rev)**	$\frac{1}{2}$	1	1	2	2
**Scalability**	$\frac{1}{2}$	1	1	1	1
**Integration Effort (rev)**	$\frac{1}{2}$	$\frac{1}{2}$	1	1	1
**TCO (rev)**	$\frac{1}{2}$	$\frac{1}{2}$	1	1	1

Notes: Numbers follow the 1–9 Saaty scale. Reciprocals are written as fractions for clarity.

TABLE VI
DERIVED AHP WEIGHTS AND CONSISTENCY DIAGNOSTICS

**Criterion**	**Weight**
Accuracy	0.329
Latency (rev)	0.221
Scalability	0.165
Integration Effort (rev)	0.143
TCO (rev)	0.143
$\lambda_{\max}$	5.078
CI	0.019
CR ($\mathrm{RI}_5 = 1.12$)	0.017

$\mathrm{CR} < 0.10$ indicates acceptable consistency.

TABLE VII
ILLUSTRATIVE NORMALISED CRITERION SCORES $r_{s,j}$ AND AGGREGATED SCORES

	Acc	Lat (rev)	Sca	IE (rev)	TCO (rev)	**Score**
Rule-based	74	69	66	64	60	68.2
Expert system	80	75	72	68	66	73.9
Reinforcement learning	94	90	86	78	83	**87.9**

Weights from Table VI applied; illustrative only

TABLE VIII
SENSITIVITY OF SYSTEM SCORES TO $\pm 10\%$ WEIGHT PERTURBATIONS

**Perturbed**	$\pm$	**RL**	**Expert**	**Rule**
Accuracy	+10%	+0.19	+0.20	+0.19
	−10%	−0.21	−0.21	−0.20
Latency (rev)	+10%	+0.04	+0.02	+0.02
	−10%	−0.05	−0.03	−0.02
Scalability	+10%	−0.03	−0.03	−0.03
	−10%	+0.03	+0.03	+0.04
Integration Effort (rev)	+10%	−0.14	−0.08	−0.06
	−10%	+0.14	+0.08	+0.06
TCO (rev)	+10%	−0.07	−0.11	−0.11
	−10%	+0.07	+0.11	+0.12

Interpretation: Positive values indicate the system benefits when that criterion becomes more prominent. RL remains top-ranked across plausible weight shifts.
